# Supplementary material for: Genetic and functional analysis of unproductive splicing using LeafCutter2
Source: bioRxiv. 2025 Apr 8:2025.04.06.646893. Preprint. [Version 1] doi: 10.1101/2025.04.06.646893 (PMC12026817; doi:10.1101/2025.04.06.646893)
Supplement: Supplement 5 [file NIHPP2025.04.06.646893v1-supplement-5.pdf]

bioRxiv preprint doi: https://doi.org/10.1101/2025.04.06.646893; this version posted April 8, 2025. The copyright holder for this preprint (which was not certified by peer review) is the author/funder, who has granted bioRxiv a license to display the preprint in perpetuity. It is made available under aCC-BY 4.0 International license.

Supplementary figures

bioRxiv preprint doi: https://doi.org/10.1101/2025.04.06.646893; this version posted April 8, 2025. The copyright holder for this preprint (which was not certified by peer review) is the author/funder, who has granted bioRxiv a license to display the preprint in perpetuity. It is made available under aCC-BY 4.0 International license.

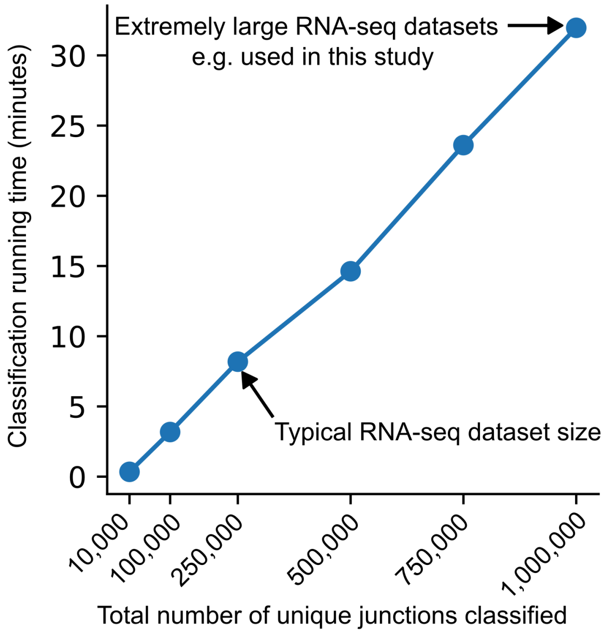

Supplementary Figure 1. LeafCutter2's junction classifier runtime as a function of the total number of splice junctions. Most datasets are expected to take approximately 10 minutes longer to be processed compared to the original LeafCutter pipeline. Large datasets with one million splice junctions will take 30 minutes longer.

bioRxiv preprint doi: https://doi.org/10.1101/2025.04.06.646893; this version posted April 8, 2025. The copyright holder for this preprint (which was not certified by peer review) is the author/funder, who has granted bioRxiv a license to display the preprint in perpetuity. It is made available under aCC-BY 4.0 International license.

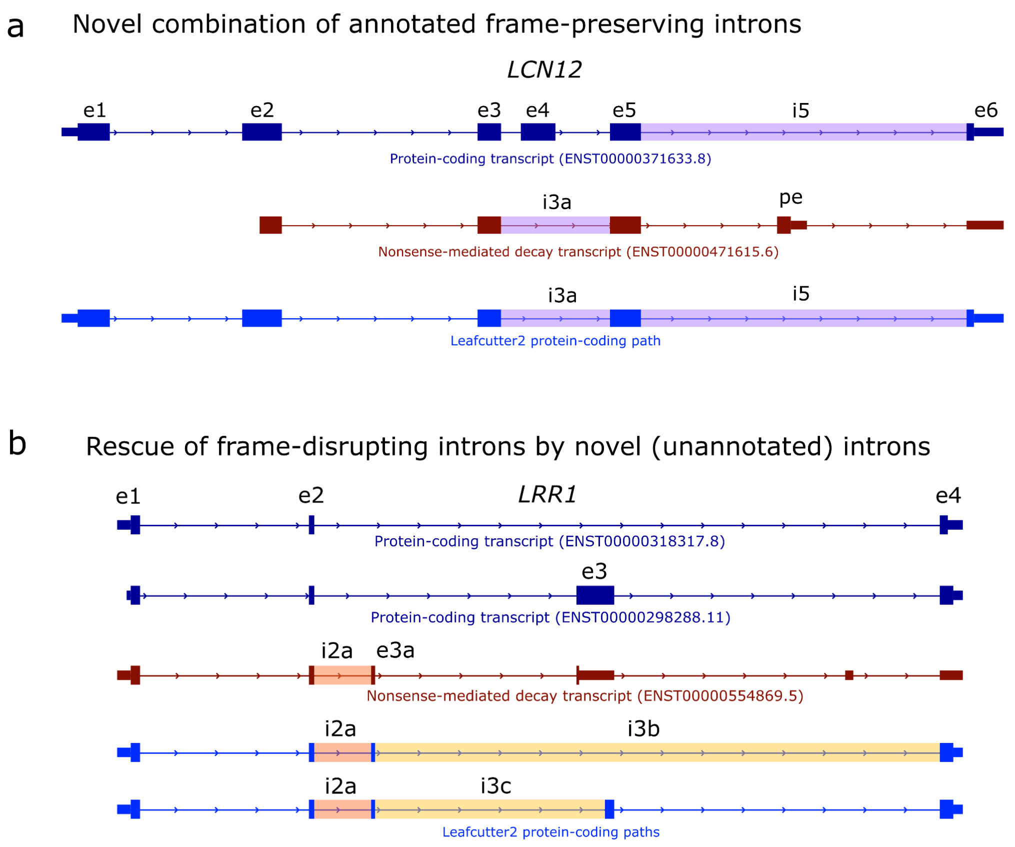

Supplementary Figure 2. Examples of splice junctions exclusively annotated in Gencode NMD transcripts, but classified as productive by LeafCutter2. a) The splice junction (i3a) skipping exon 4 (e4) in *LCN12* is only annotated in the transcript ENST0000047165, which is annotated as a nonsense-mediated decay transcript. However, the premature termination codon in this transcript comes from a downstream poison exon (pe). Since e4 is a symmetric exon (its length is a multiple of 3), i3a does not disrupt the reading frame, which allows LeafCutter2 to find a novel protein-coding path between the start codon in exon 1 (e1) and the stop codon in exon 6 (e6). **b)** The introduction of an alternative exon (e3a) between protein coding exons 2 (e2) and 3 (e3) in *LRR1* disrupts the open reading frame. This creates a premature termination codon in e3, creating a premature termination codon, which targets transcript ENST00000554869 for nonsense-mediated decay. As a result, the splice junction i2a is annotated as unproductive in Gencode. However, two splice junctions (i3b and i3c) missing from the annotation but present in nascent RNA LCL data[1] can rescue the reading frame disruption caused by i2a, creating a protein coding path between the start codon in exon 1 (e1) and the stop codon in exon 4 (e4)

bioRxiv preprint doi: https://doi.org/10.1101/2025.04.06.646893; this version posted April 8, 2025. The copyright holder for this preprint (which was not certified by peer review) is the author/funder, who has granted bioRxiv a license to display the preprint in perpetuity. It is made available under aCC-BY 4.0 International license.

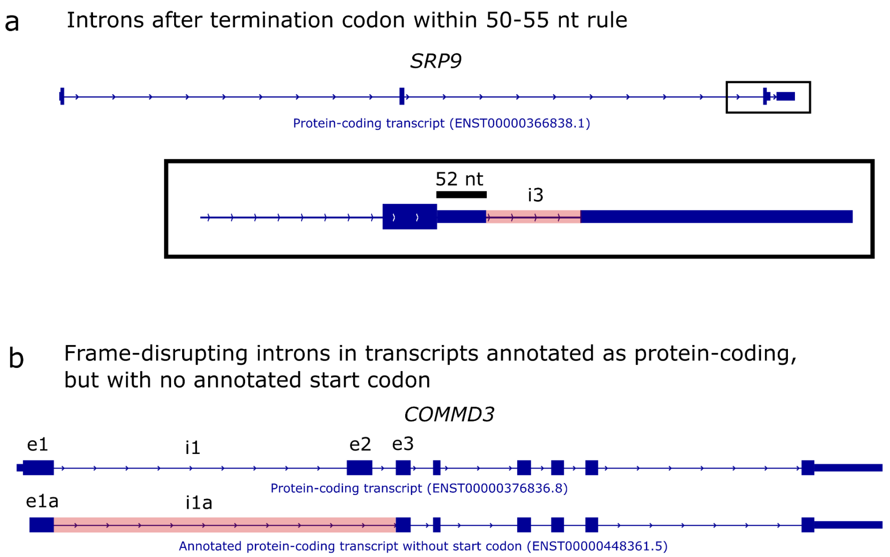

Supplementary Figure 3. Examples of splice junctions in Gencode protein coding transcripts that are classified as unproductive by LeafCutter2. a) Splice junction i3 in *STP9* falls after the stop codon on the last coding exon, but within the 50-55 nucleotide rule. LeafCutter2 misclassifies this splice junction as unproductive. **b)** Transcript ENST00000448361 in *COMMD3* is annotated as protein-coding, but its Gencode annotation lacks a start codon. Intron i1a in this transcript skips exon 2 (e2), an asymmetric exon (length is not a multiple of 3) in other protein-coding transcripts with annotated start and stop codons. Because of the missing start codon and the reading frame disruption, LeafCutter2 is unable to find a viable protein-coding path that includes splice junction i1a.

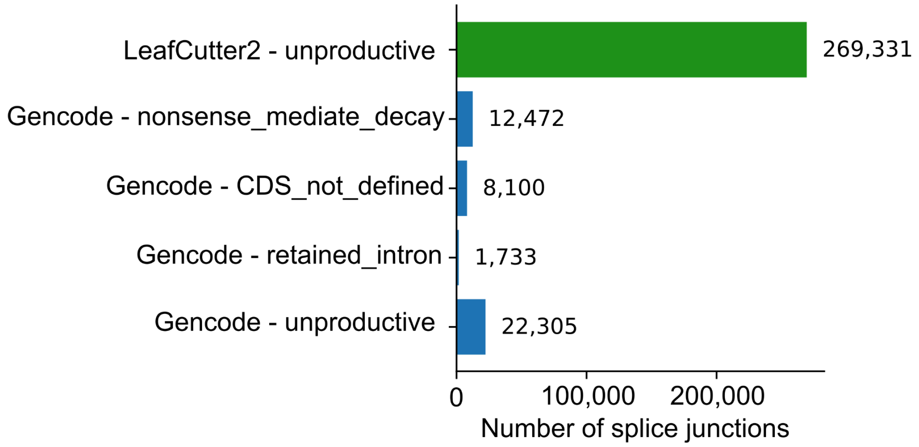

Supplementary Figure 4. LeafCutter2 finds hundreds of thousands novel unproductive splice junctions in our dataset of 86 nascent RNA samples across 86 Lymphoblastoid cell lines. Most of these are novel. In contrast, Gencode only has 22,305 unproductive splice junctions across multiple categories.

bioRxiv preprint doi: https://doi.org/10.1101/2025.04.06.646893; this version posted April 8, 2025. The copyright holder for this preprint (which was not certified by peer review) is the author/funder, who has granted bioRxiv a license to display the preprint in perpetuity. It is made available under aCC-BY 4.0 International license.

Correlation between RNA Integrity Number and percentage of unproductive splice junctions

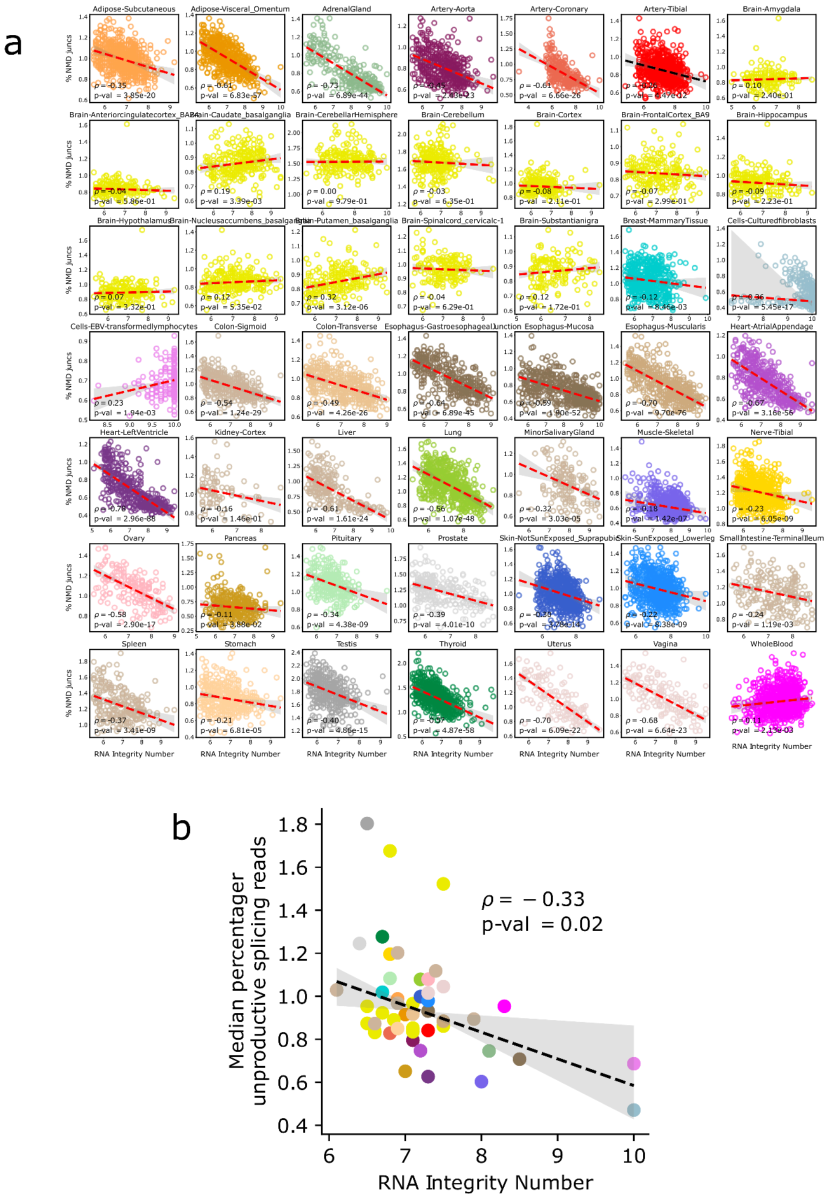

Supplementary Figure 5. Correlation between RNA Integrity Number (RIN) and percentage of unproductive splice junctions. a) Per sample within the same GTEx tissue, and **b)** median across different tissues.

bioRxiv preprint doi: https://doi.org/10.1101/2025.04.06.646893; this version posted April 8, 2025. The copyright holder for this preprint (which was not certified by peer review) is the author/funder, who has granted bioRxiv a license to display the preprint in perpetuity. It is made available under aCC-BY 4.0 International license.

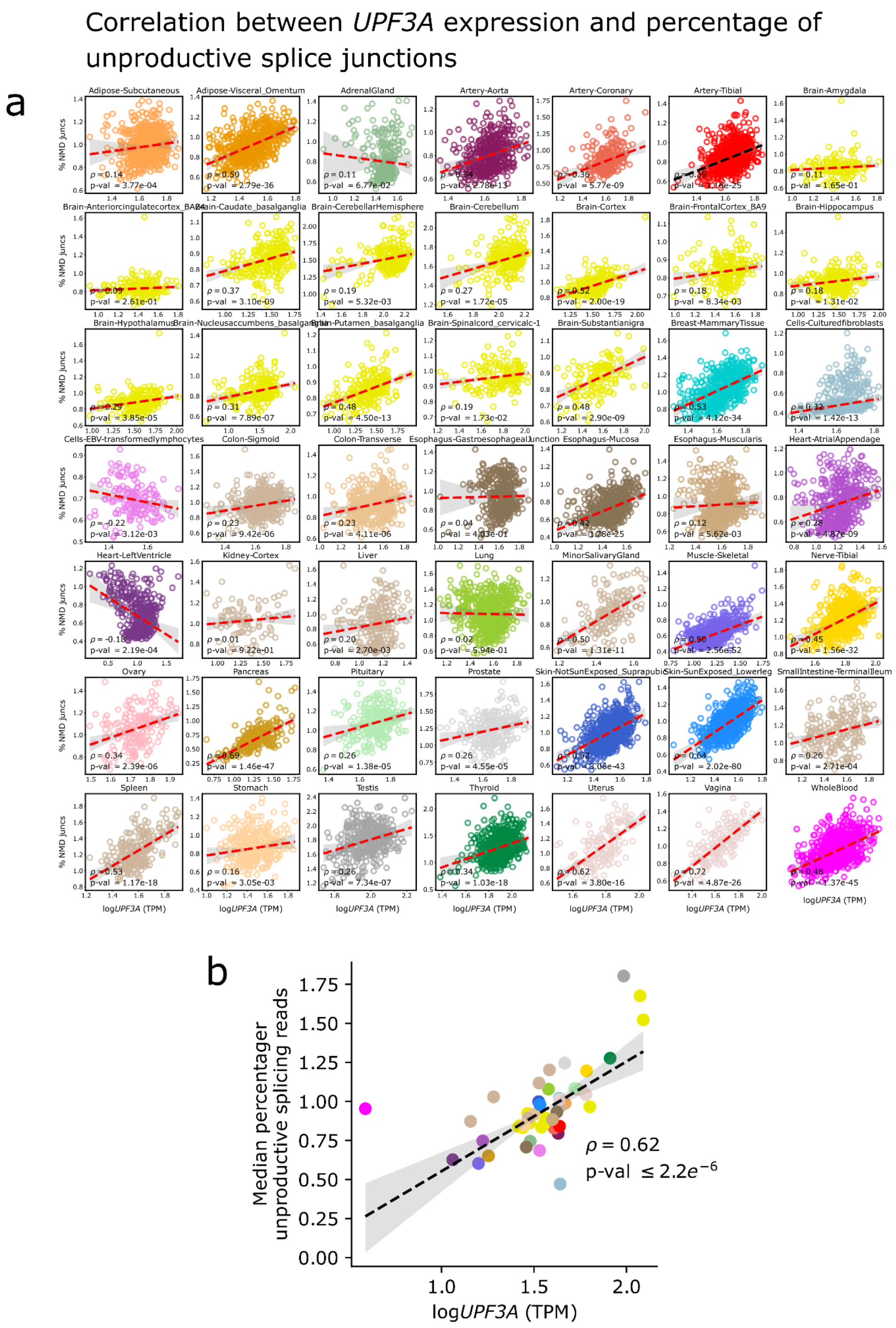

Supplementary Figure 6. Correlation between *UPF3A* expression and percentage of unproductive splice junctions. a) Per sample within the same GTEx tissue, and **b)** median across different tissues.

bioRxiv preprint doi: https://doi.org/10.1101/2025.04.06.646893; this version posted April 8, 2025. The copyright holder for this preprint (which was not certified by peer review) is the author/funder, who has granted bioRxiv a license to display the preprint in perpetuity. It is made available under aCC-BY 4.0 International license.

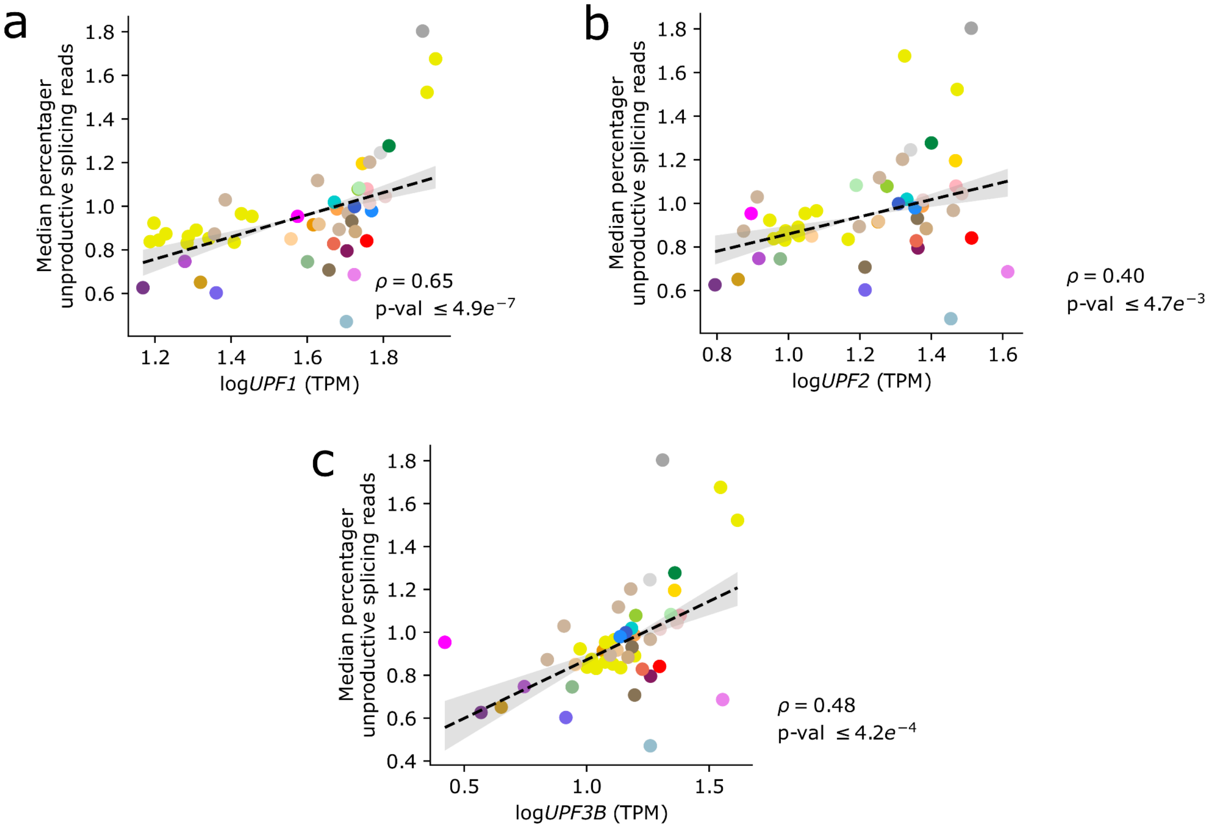

Supplementary Figure 7. Correlation between NMD factors expression and percentage of unproductive splice junctions. a) *UPF1*, **b)** *UPF2*, **c)** *UPF3B*.

bioRxiv preprint doi: https://doi.org/10.1101/2025.04.06.646893; this version posted April 8, 2025. The copyright holder for this preprint (which was not certified by peer review) is the author/funder, who has granted bioRxiv a license to display the preprint in perpetuity. It is made available under aCC-BY 4.0 International license.

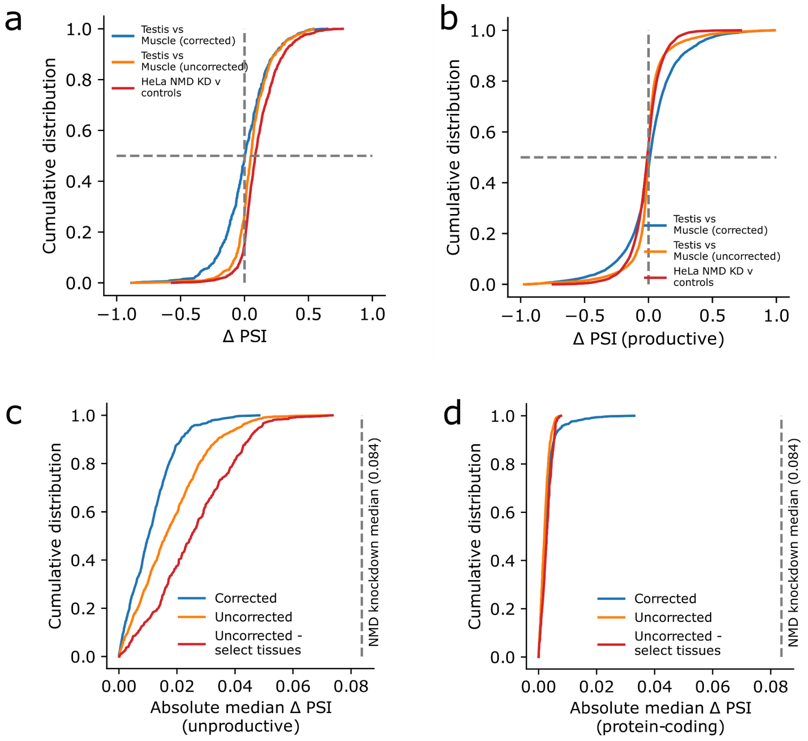

Supplementary Figure 8. Effect of NMD efficiency and covariate correction in LeafCutter's differential splicing test. (a) Distribution of delta PSI of significant unproductive splice junctions between testis and skeletal muscle when correcting for the percentage of unproductive splice junctions (blue), without correction (orange), and between HeLa cell lines with NMD factors *SMG6/SMG7* double shRNA knockdown (dKD) and controls (red). The dKD versus control comparison serves as an example of contrast that reflects only differences in NMD activity rather than cell-type-specific regulation of unproductive splicing. These data indicate that correcting for the percentage of unproductive splice junctions is required and sufficient to measure unbiased cell-type-specific regulation of unproductive splicing. **(b)** Distribution of delta PSI for productive splice junctions between the same groups. **(c)** Absolute deviation from 0 of the median of the delta PSI distribution of unproductive splice junctions across all tissue pairs when correcting for the percentage of unproductive splice junctions (blue), without correction (orange), and uncorrected when the pairwise comparison contains one of the top three (Testis, Brian - Cerebellum, Brain - Cerebellar Hemisphere) or bottom three (Heart - Left Ventricle, Muscle - Skeletal, Cells - Cultured Fibroblasts) tissues regarding their percentage of unproductive splice junctions (red). **(d)** Absolute deviation from 0 of the delta PSI distribution of productive splice junctions across the same groups. These observations again suggest that differences in NMD activity bias differential splicing measurements of unproductive splicing events, but can be corrected using *UPF3A* expression level as covariate.

bioRxiv preprint doi: https://doi.org/10.1101/2025.04.06.646893; this version posted April 8, 2025. The copyright holder for this preprint (which was not certified by peer review) is the author/funder, who has granted bioRxiv a license to display the preprint in perpetuity. It is made available under aCC-BY 4.0 International license.

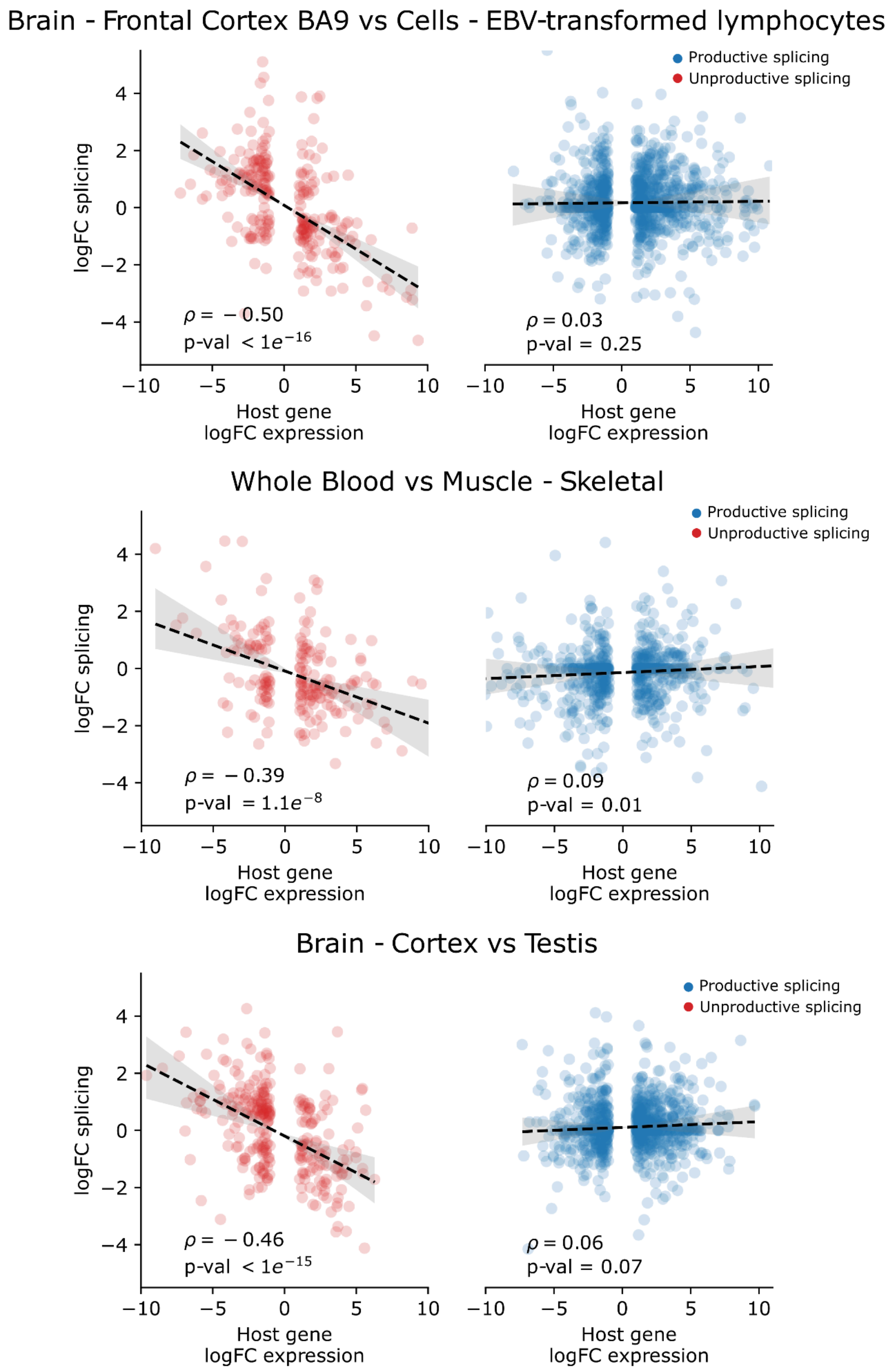

Supplementary Figure 9. Examples of the negative correlation differential unproductive splicing and differential gene expression across tissue pairs in GTEx. Differential productive splicing has little to no correlation with differential gene expression.

bioRxiv preprint doi: https://doi.org/10.1101/2025.04.06.646893; this version posted April 8, 2025. The copyright holder for this preprint (which was not certified by peer review) is the author/funder, who has granted bioRxiv a license to display the preprint in perpetuity. It is made available under aCC-BY 4.0 International license.

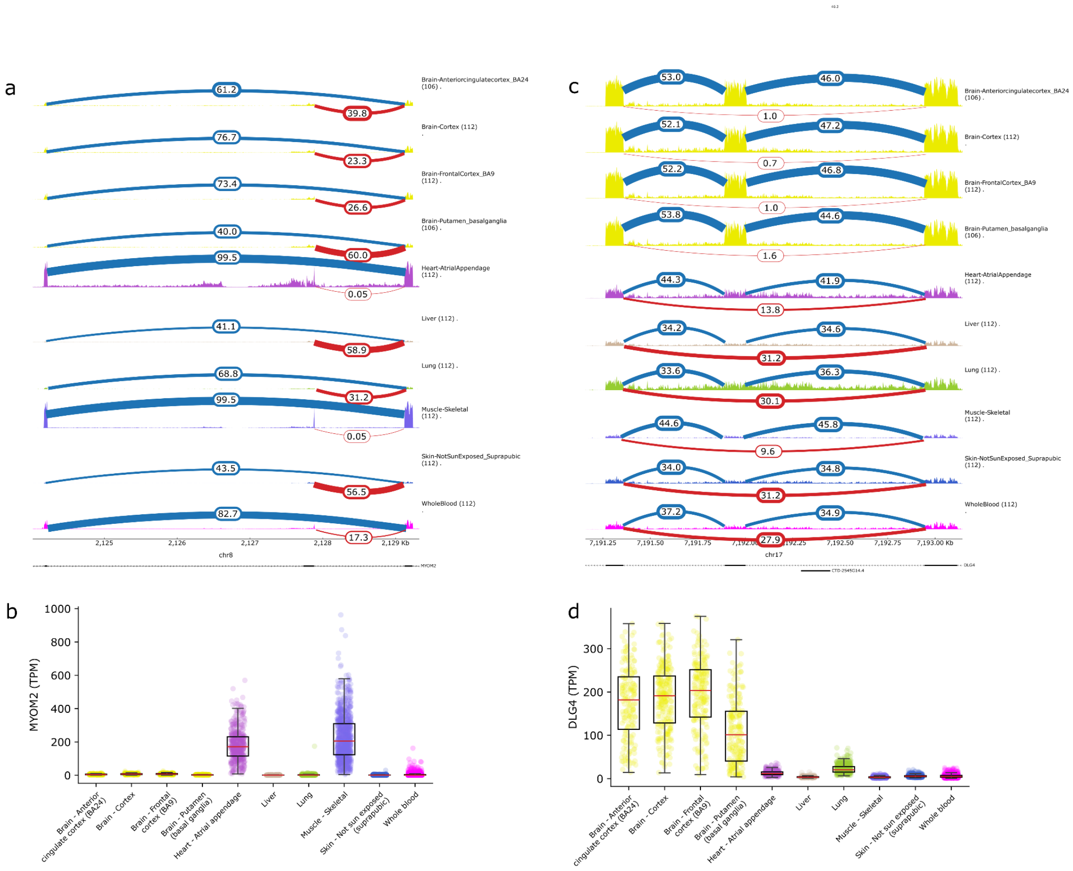

Supplementary Figure 10. Examples of differential unproductive splicing and its association with gene expression. a) Unproductive splicing in *MYOM2* is relatively high in most tissues except skeletal muscle and heart tissue. **b)** *MYOM2* is primarily expressed in skeletal muscle and heart tissues. **c)** Unproductive splicing in *DLG4* is low in brain tissues, but relatively elevated in all other tissues. **d)** *DLG4* is primarily expressed in brain tissues.

bioRxiv preprint doi: https://doi.org/10.1101/2025.04.06.646893; this version posted April 8, 2025. The copyright holder for this preprint (which was not certified by peer review) is the author/funder, who has granted bioRxiv a license to display the preprint in perpetuity. It is made available under aCC-BY 4.0 International license.

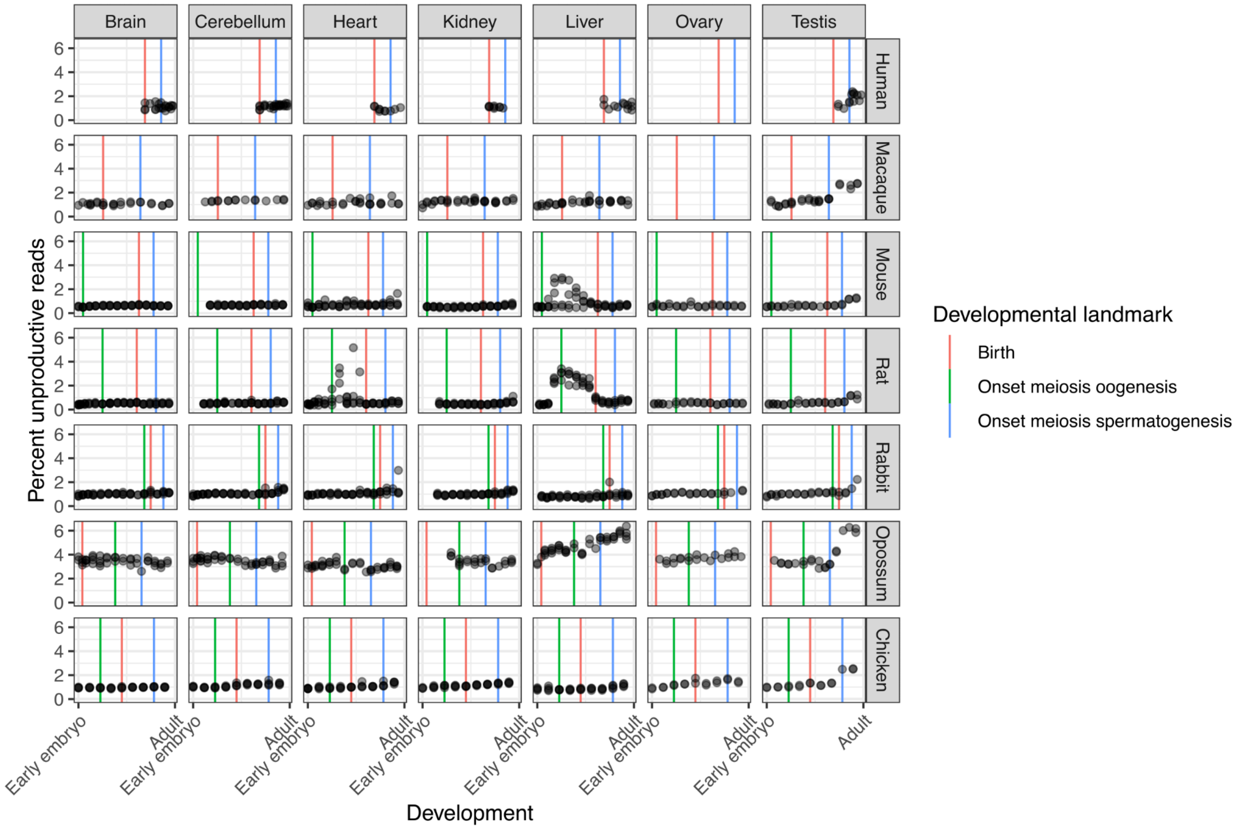

Supplementary Figure 11. Percent of unproductive reads across species, tissues, and developmental time. RNA-seq samples (points) are ordered by developmental stage within each tissue and species, and the percentage of splice junction reads which are classified as unproductive is plotted. Because samples across species do not necessarily reflect equivalent developmental time courses, we also included developmental landmarks for each of the developmental time courses in each species, as defined by the source publication[38].

bioRxiv preprint doi: https://doi.org/10.1101/2025.04.06.646893; this version posted April 8, 2025. The copyright holder for this preprint (which was not certified by peer review) is the author/funder, who has granted bioRxiv a license to display the preprint in perpetuity. It is made available under aCC-BY 4.0 International license.

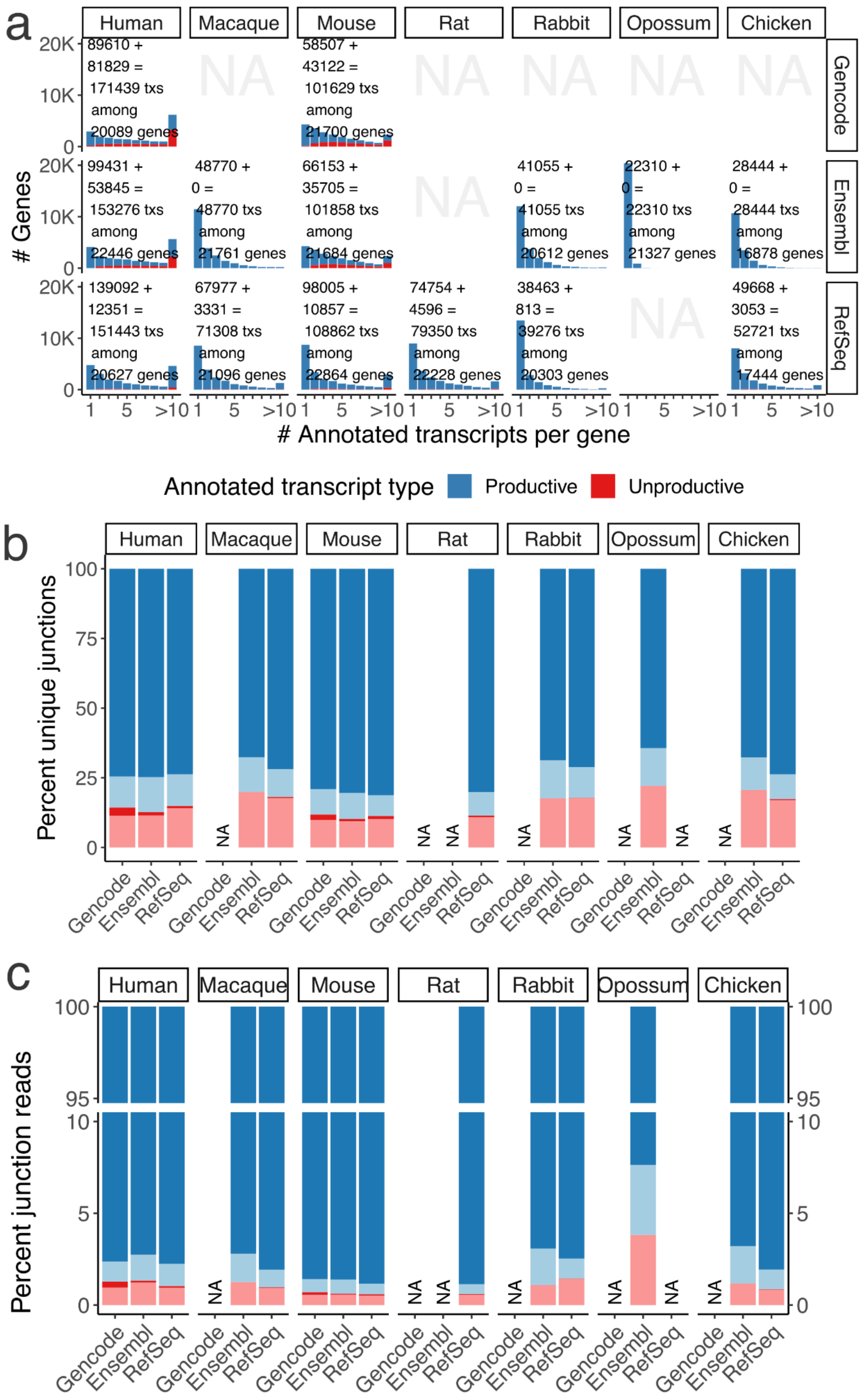

bioRxiv preprint doi: https://doi.org/10.1101/2025.04.06.646893; this version posted April 8, 2025. The copyright holder for this preprint (which was not certified by peer review) is the author/funder, who has granted bioRxiv a license to display the preprint in perpetuity. It is made available under aCC-BY 4.0 International license.

Supplementary Figure 12. Various sources of gene annotations yield similar fractions of unproductive splice junctions and transcripts. a) Histograms quantify the number of transcripts per protein-coding gene in each species' current gene annotation. The fraction of transcripts that are productive are filled as a fraction within each bin. For each species, gene annotations can be sourced from one or more sources (ie, Gencode, Ensembl, or RefSeq. Some gene annotations not available (NA) from either Gencode or UCSC (which itself sources annotations from Ensembl and/or RefSeq). **b**) Percent of unique junctions (with a minimum abundance of 0.05 junction reads per million junction reads) that are productive or unproductive, and, annotated or unannotated across gene annotation sources. All samples (all tissues and all post-birth developmental time points) are combined for simplicity. **c**) Similar to B, but plotting the percent of splice junction *reads* in each category

bioRxiv preprint doi: https://doi.org/10.1101/2025.04.06.646893; this version posted April 8, 2025. The copyright holder for this preprint (which was not certified by peer review) is the author/funder, who has granted bioRxiv a license to display the preprint in perpetuity. It is made available under aCC-BY 4.0 International license.

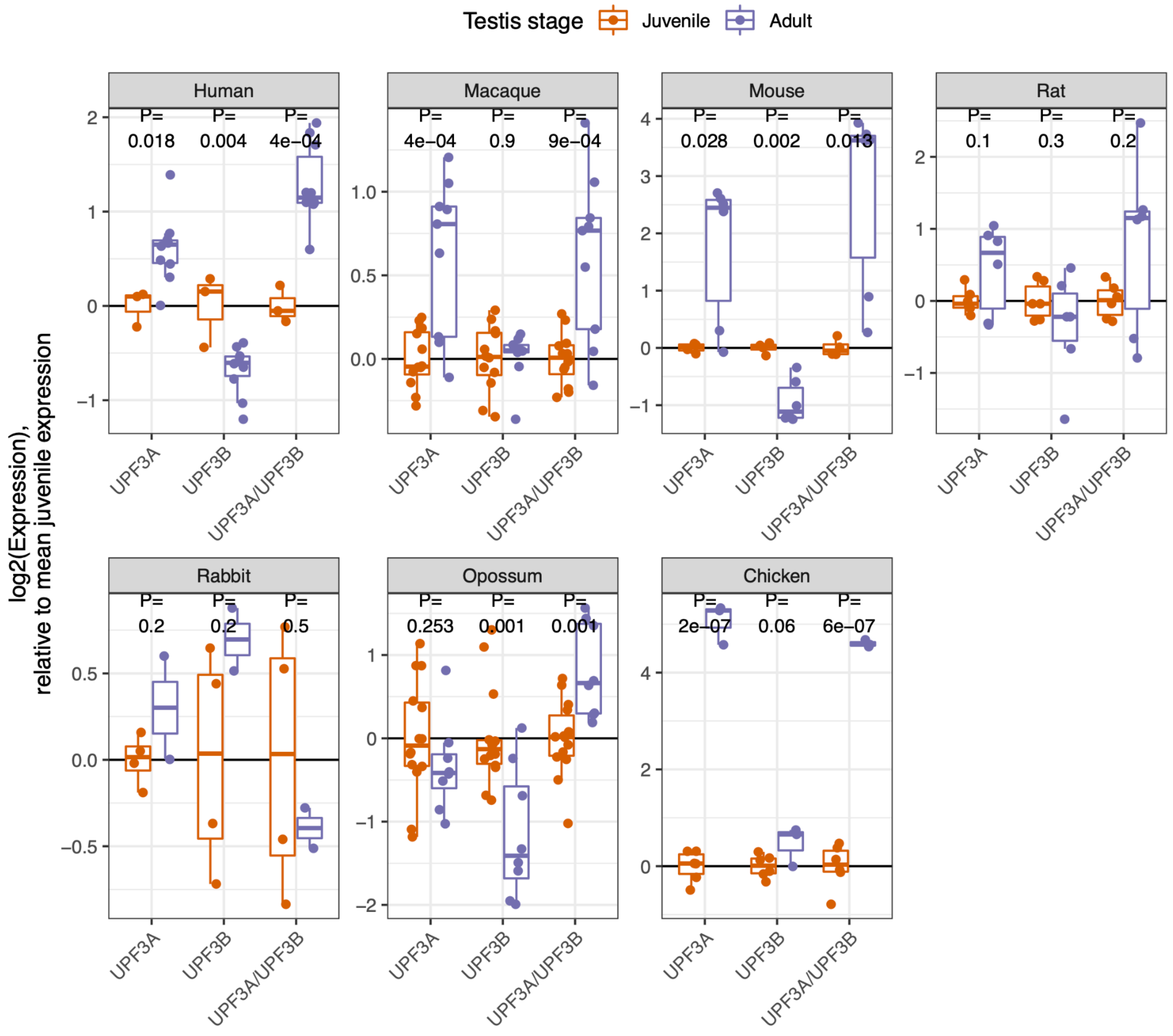

Supplementary Figure 13. *UPF3A* and *UPF3B* expression across testis development. Boxplots (inner quartiles with whiskers that extend to the most extreme value within 1.5 times the interquartile range from the hinge) and points for individual samples depict the relative expression (RNA-seq) of *UP3A*, *UPF3B*, and the *UPF3A*/*UPF3B* ratio in juvenile and adult testis in each species. P-values from two-sided t-test.

bioRxiv preprint doi: https://doi.org/10.1101/2025.04.06.646893; this version posted April 8, 2025. The copyright holder for this preprint (which was not certified by peer review) is the author/funder, who has granted bioRxiv a license to display the preprint in perpetuity. It is made available under aCC-BY 4.0 International license.

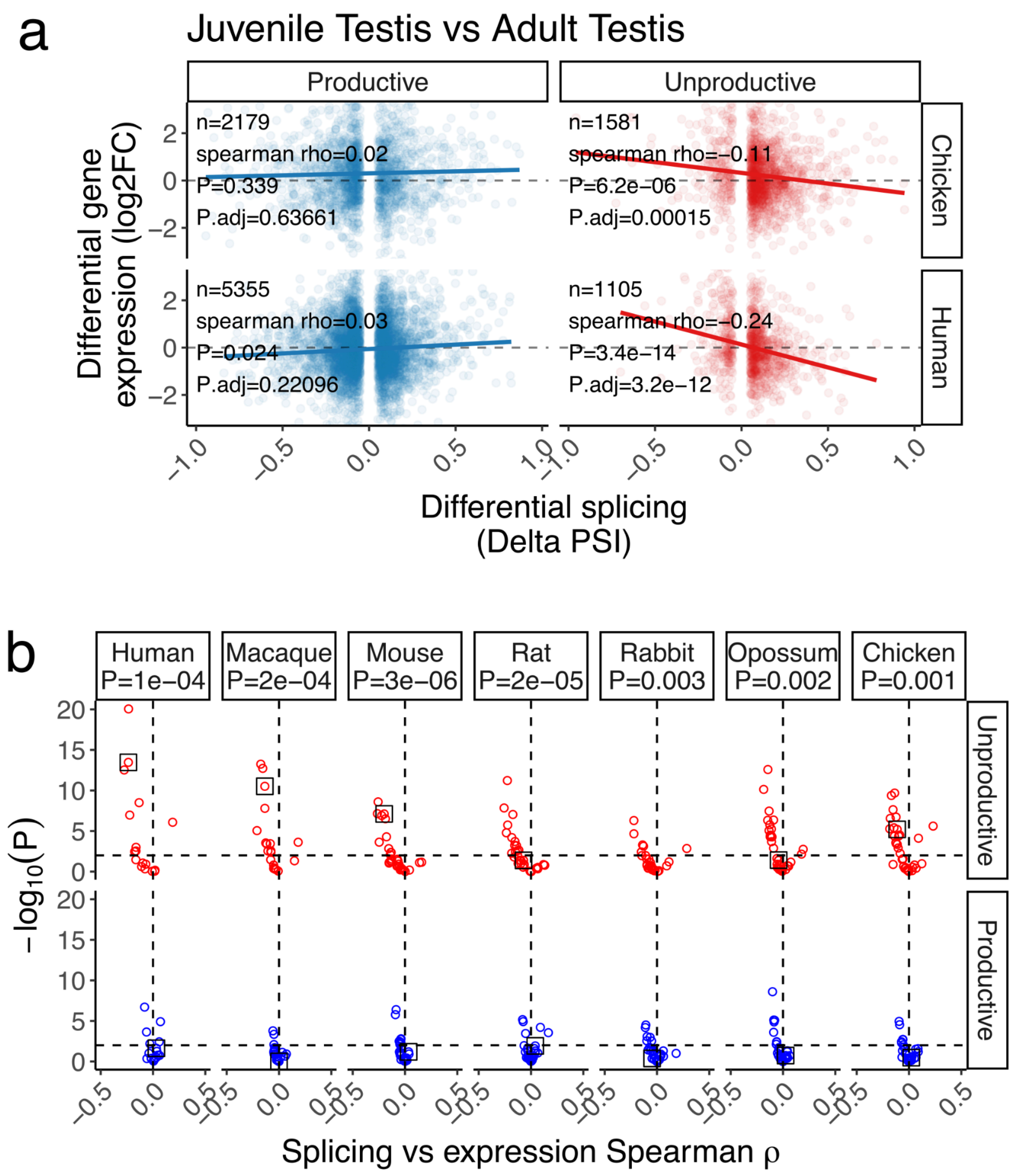

Supplementary Figure 14. Correlation between splicing and expression for unproductive and productive differentially spliced junctions. a) Scatter plots showing the Spearman's correlation between splicing (delta PSI) and differential gene expression (logFoldChange) for significant differential splicing events (FDR<0.05% and delta PSI > 5%) between juvenile and adult testis in human, grouped by whether the splice event is productive or unproductive. A similar contrast is shown for chicken. P values for spearman correlation coefficient, and adjusted for multiple test correction. **b)** for all contrasts (including comparisons between adult tissue type within each species, and different developemental timepoints within each tissue within each species, see Methods) and the significance and correlation coefficient of the splicing vs expression is shown. Only well powered contrasts with at least 100 significant differentially spliced intron clusters were included. The contrast for juvenile vs adult testis for each species is highlighted with a black box around the point. P-values correspond to a one-sided Mann-Whitney test, comparing the distribution of spearman coefficients for contrasts among productive vs unproductive junctions.

bioRxiv preprint doi: https://doi.org/10.1101/2025.04.06.646893; this version posted April 8, 2025. The copyright holder for this preprint (which was not certified by peer review) is the author/funder, who has granted bioRxiv a license to display the preprint in perpetuity. It is made available under aCC-BY 4.0 International license.

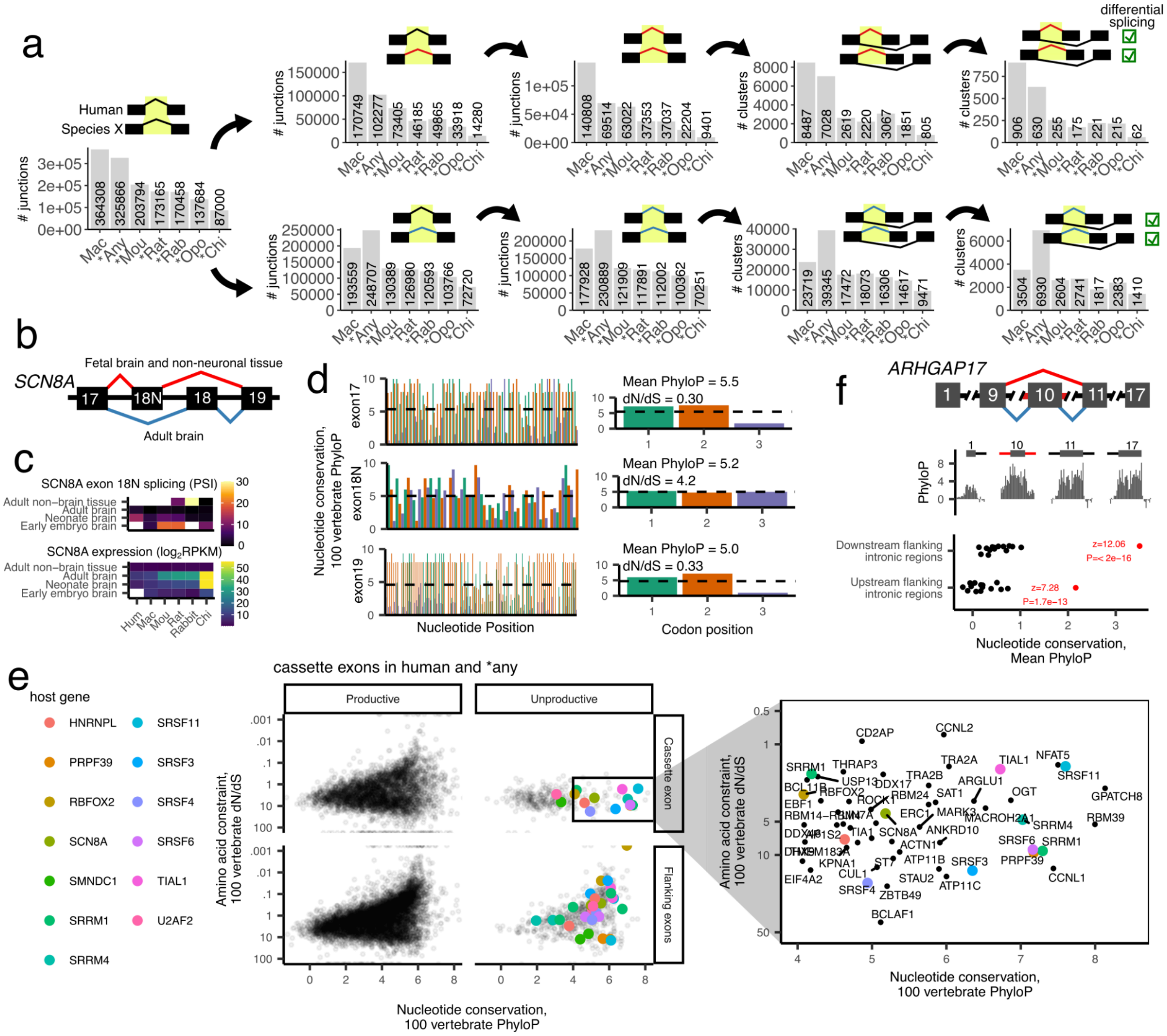

Supplementary Figure 15. Identification of evolutionarily conserved unproductive splicing events. a) Splice events classified as conserved between human and each query species by series of filters. Number splice events (splice junctions, or unique clusters containing at least one splice junction) passing each filter in query species depicted as bars. *Any refers to any non-macaque query species. Filters are: 1) Query-species splice junction is mapped with liftover to splice junction observed in human, 2) splice junction is unproductive in query species, 3) and unproductive in human, 4) and is alternatively spliced in both, 5) in a tissue- or developmental-specific fashion as assessed by leafcutter differential splicing (Methods for details). Filters 2-5 similarly applied for productive splice events (lower row). **b**) Alternative splicing gene-structure of poison exon in SCNA8. **c**) Mean expression and splicing of SCN8A poison exon across samples from various tissues and brain development stages. **d**) Nucleotide conservation (100 vertebrate PhyloP score) across the *SCN8A* poison exon and flanking exons, colored by codon position. Average PhyloP by codon position, and amino acid constraint (dN/dS) plotted on the right for each exon. **e**) Nucleotide conservation and amino acid constraint

bioRxiv preprint doi: https://doi.org/10.1101/2025.04.06.646893; this version posted April 8, 2025. The copyright holder for this preprint (which was not certified by peer review) is the author/funder, who has granted bioRxiv a license to display the preprint in perpetuity. It is made available under aCC-BY 4.0 International license.

for unproductive (poison) and productive exons present in human and *any query species (passes filter 4). Each point is an exon. Unproductive cassette exons in genes of interest colored, or labelled in zoomed inset to highlight poison exons with high nucleotide conservation but low amino acid conservation. **f**) Gene structure (top) for unproductive exon skipping junction in ARHGAP17. Nucleotide conservation for 100bp intronic splice site regions (black) flanking each of the ARHGAP17’s 17 exons, highlighting intronic regions flanking the skipped exon (red). P-value represents a two-sided z-test.

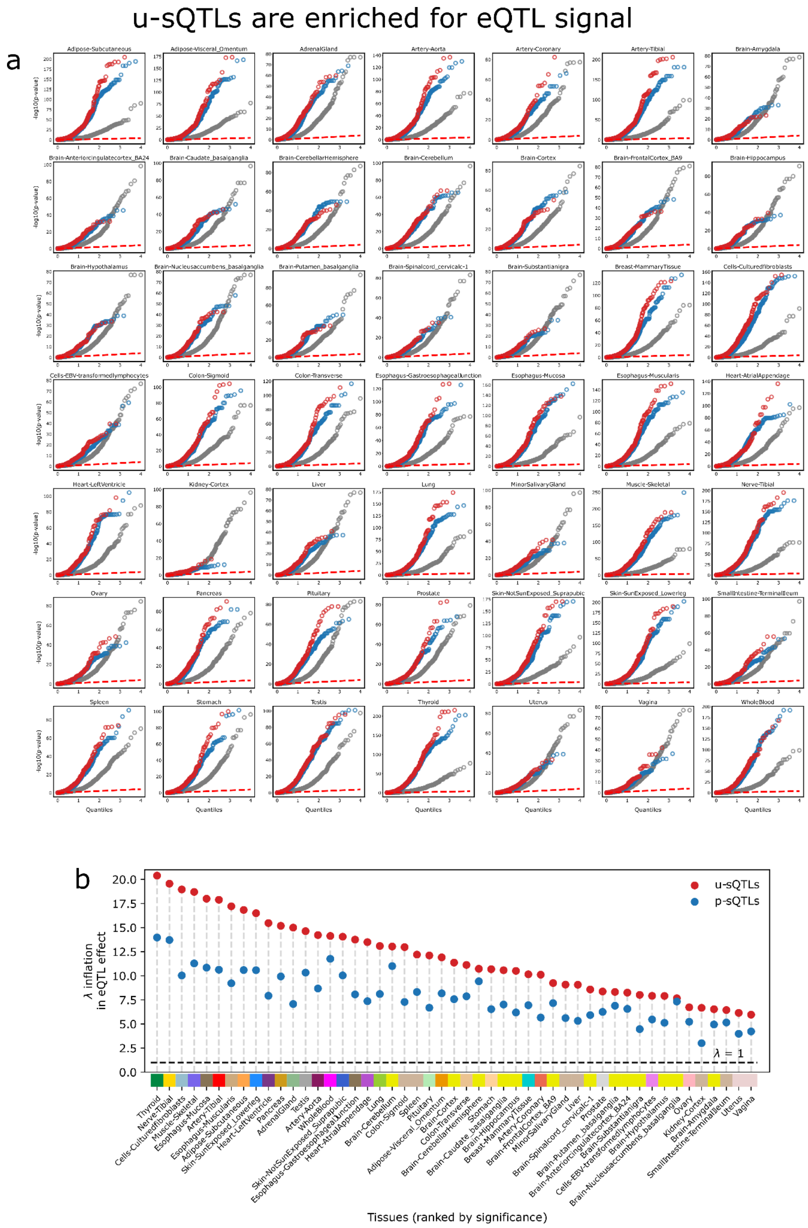

Supplementary Figure 16. Enrichment of eQTL signals in u-sQTLs. a) QQ plots of the eQTL effect across 49 GTEx tissues of u-sQTLs (red), p-sQTLs (blue) and random SNPs (gray). **b)**

bioRxiv preprint doi: https://doi.org/10.1101/2025.04.06.646893; this version posted April 8, 2025. The copyright holder for this preprint (which was not certified by peer review) is the author/funder, who has granted bioRxiv a license to display the preprint in perpetuity. It is made available under aCC-BY 4.0 International license.

Lambda inflation shows enrichment in the eQTL p-value distributions of sQTLs, with u-sQTLs signals showing more strength than p-sQTLs across all GTEx tissues.

Variant associated with hypothyroidism affects unproductive splicing in thyroid and other relevant tissues.

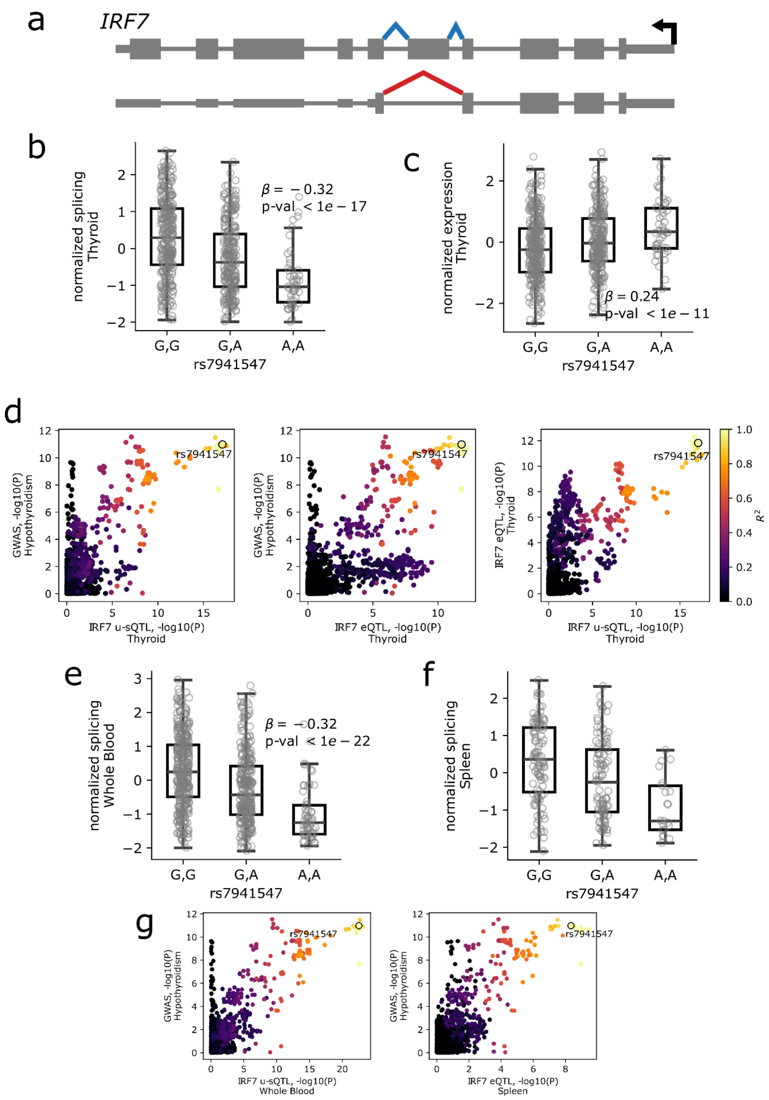

Supplementary Figure 17. Hypothyroidism variant in *IRF7* is associated with unproductive splicing and gene expression changes. a) Productive-unproductive alternative splicing event in *IRF7*. **b)** Variant rs7941547, strongly associated with hypothyroidism, has a strong effect on *IRF7* unproductive splicing in Thyroid tissue. **c)** Variant rs7941547 has a strong effect on *IRF7* expression in a direction consistent with AS-NMD in Thyroid tissue. **d)** LocusCompare plots between Hypothyroidism GWAS signal, u-sQTL effect in IRF7 in Thyroid

bioRxiv preprint doi: https://doi.org/10.1101/2025.04.06.646893; this version posted April 8, 2025. The copyright holder for this preprint (which was not certified by peer review) is the author/funder, who has granted bioRxiv a license to display the preprint in perpetuity. It is made available under aCC-BY 4.0 International license.

tissue, and eQTL effect in *IRF7* in Thyroid tissue. **e)** Variant rs7941547 has a strong effect on *IRF7* unproductive splicing in other potentially relevant tissues such as Whole Blood and f) Spleen. **g)** LocusCompare plots between Hypothyroidism GWAS signal, u-sQTL effect in *IRF7* in Whole Blood and Spleen tissue.

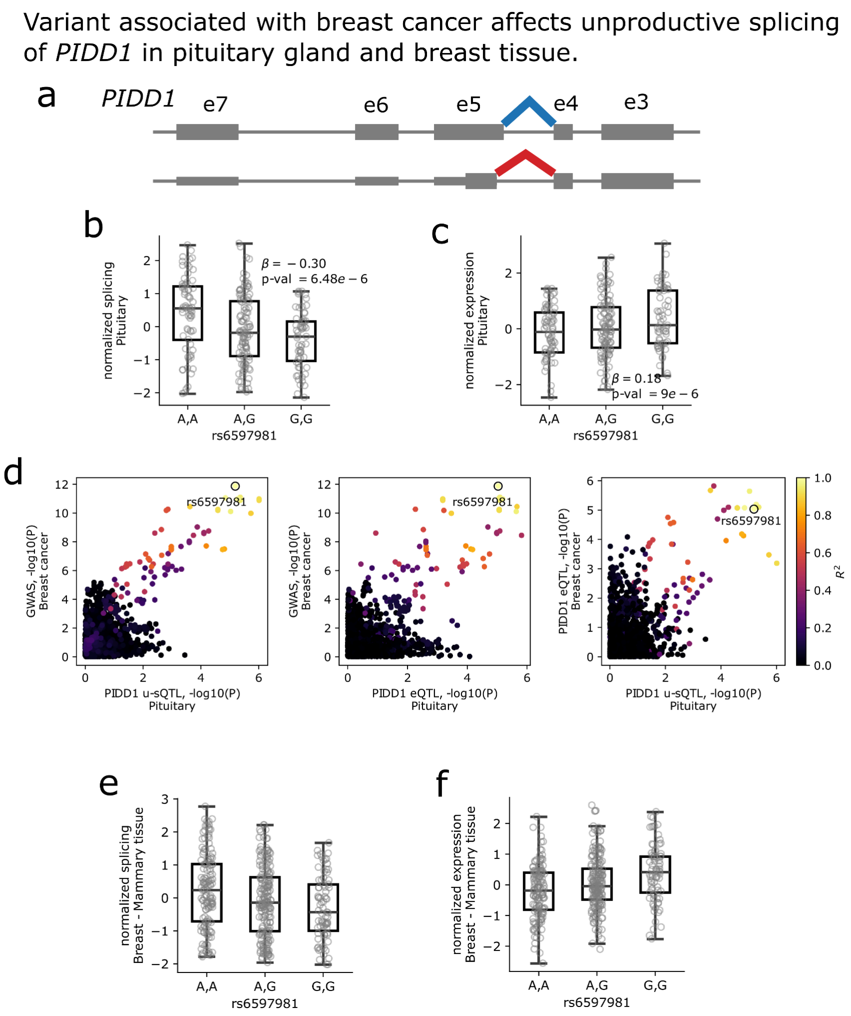

Supplementary Figure 18. Breast cancer variant in *PIDD1* is associated with unproductive splicing and gene expression changes. a) Productive-unproductive alternative splicing event in *PIDD1*. **b)** Variant rs6597981, strongly associated with breast cancer, has a strong effect on *PIDD1* unproductive splicing in Pituitary tissue. **c)** Variant rs6597981 has a strong effect on *PIDD7* expression in a direction consistent with AS-NMD in Pituitary tissue. **d)** LocusCompare plots between Hypothyroidism GWAS signal, u-sQTL effect in *PIDD1* in Pituitary tissue, and eQTL effect in PIDD1 in Pituitary tissue. **e)** Variant rs6597981 has an effect on *PIDD1* unproductive splicing and f) expression in breast mammary tissue. These effects are consistent with the observations in Pituitary tissue, but the u-sQTL effect barely misses the significance threshold.

bioRxiv preprint doi: https://doi.org/10.1101/2025.04.06.646893; this version posted April 8, 2025. The copyright holder for this preprint (which was not certified by peer review) is the author/funder, who has granted bioRxiv a license to display the preprint in perpetuity. It is made available under aCC-BY 4.0 International license.

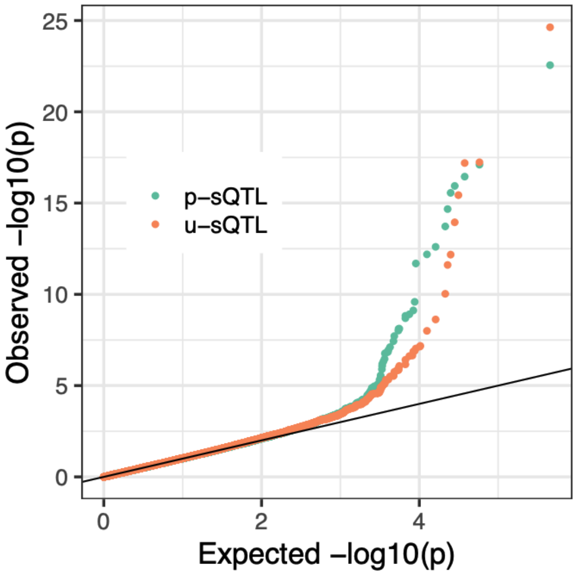

Supplementary Figure 19. Enrichment of haQTL p-values for u-sQTLs and p-sQTLs. Unproductive splicing QTLs (u-sQTLs) did not exhibit significantly different enrichment in haQTL compared to productive splicing QTLs (p-sQTLs).

bioRxiv preprint doi: https://doi.org/10.1101/2025.04.06.646893; this version posted April 8, 2025. The copyright holder for this preprint (which was not certified by peer review) is the author/funder, who has granted bioRxiv a license to display the preprint in perpetuity. It is made available under aCC-BY 4.0 International license.

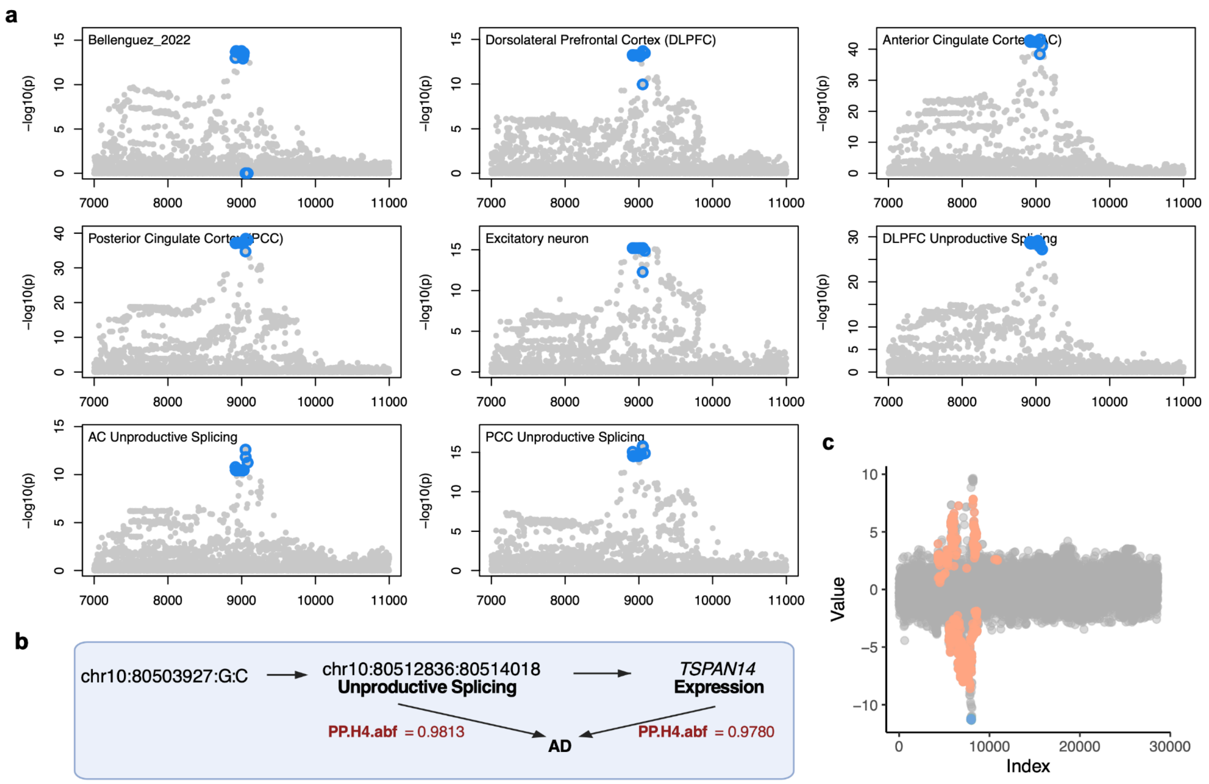

Supplementary Figure 20. ColocBoost analysis and proposed regulatory model for *TSPAN14* unproductive splicing in AD.
(a) Colocalization signals are observed among AD risk and unproductive splicing of *TSPAN14* in AC, DLPFC, and PCC brain regions (chr10:80512836–80514018), as well as *TSPAN14* gene expression and excitatory neuron (Exc) expression. (b) A proposed regulatory model suggests that genetic variants in linkage disequilibrium near chr10:80503927:G:C may drive both unproductive splicing and altered TSPAN14 expression. Colocalization posterior probabilities (PP.H4.abf in SuSiE-COLOC) with AD risk loci are shown in red text. (c) Scatter plot illustrating the Z-scores for variants associated with *TSPAN14* unproductive splicing event. Variants involved in potential mediation of unproductive splicing to expression in DLPFC are highlighted in orange (FDR < 0.05 but not the reserved direction). Blue dots denote a 95% colocalized confidence set identified by ColocBoost, which overlaps with the fine-mapping 95% credible set identified by SuSiE.

bioRxiv preprint doi: https://doi.org/10.1101/2025.04.06.646893; this version posted April 8, 2025. The copyright holder for this preprint (which was not certified by peer review) is the author/funder, who has granted bioRxiv a license to display the preprint in perpetuity. It is made available under aCC-BY 4.0 International license.

Supplementary Table 1

Tissue	Total samples	Samples with genotype	p-sQTL clusters	u-sQTLs clusters
Adipose - Subcutaneous	663	581	6490	3942
Adipose - Visceral (Omentum)	541	469	5307	3126
Adrenal Gland	258	233	2986	1582
Artery - Aorta	432	387	4637	2522
Artery - Coronary	240	213	2595	1443
Artery - Tibial	663	584	6255	3583
Brain - Amygdala	152	129	1130	579
Brain - Anterior cingulate cortex (BA24)	176	147	1634	764
Brain - Caudate (basal ganglia)	246	194	2196	1091
Brain - Cerebellar Hemisphere	215	175	2612	1618
Brain - Cerebellum	241	209	3092	1865
Brain - Cortex	255	205	2550	1343
Brain - Frontal Cortex (BA9)	209	175	2030	1012
Brain - Hippocampus	197	165	1458	766
Brain - Hypothalamus	202	170	1782	918
Brain - Nucleus accumbens (basal ganglia)	246	202	2186	1158
Brain - Putamen (basal ganglia)	205	170	1723	831
Brain - Spinal cord (cervical c-1)	159	126	1358	721
Brain - Substantianigra	139	114	995	493
Breast - Mammary Tissue	459	396	4745	2963
Cells - Cultured fibroblasts	504	483	6723	3168
Cells - EBV-transformed lymphocytes	174	147	2785	1379
Colon - Sigmoid	373	318	3954	2293
Colon - Transverse	406	368	4345	2551
Esophagus - Gastroesophageal Junction	375	330	4180	2330
Esophagus - Mucosa	555	497	5417	2968
Esophagus - Muscularis	515	465	5419	3005
Heart - Atrial Appendage	429	372	4125	2158
Heart - Left Ventricle	432	386	3250	1554
Kidney - Cortex	85	73	629	332
Liver	226	208	1766	1043
Lung	578	515	5911	3598
Minor Salivary Gland	162	144	1998	1119
Muscle - Skeletal	803	706	6146	3024
Nerve - Tibial	619	532	6532	4131
Ovary	180	167	2202	1421
Pancreas	328	305	2724	1595
Pituitary	283	237	3346	2057

bioRxiv preprint doi: https://doi.org/10.1101/2025.04.06.646893; this version posted April 8, 2025. The copyright holder for this preprint (which was not certified by peer review) is the author/funder, who has granted bioRxiv a license to display the preprint in perpetuity. It is made available under aCC-BY 4.0 International license.

Prostate	245	221	2679	1800
Skin - Not Sun Exposed (Suprapubic)	604	517	5639	3417
Skin - Sun Exposed (Lower leg)	701	605	6353	3782
Small Intestine - Terminal Ileum	187	174	2281	1368
Spleen	241	227	3082	1870
Stomach	359	324	3261	1894
Testis	361	322	7107	4589
Thyroid	653	574	6985	4169
Uterus	142	129	1814	1072
Vagina	156	141	1746	1082
Whole Blood	755	670	4080	2413
**All tissues**	**17,329**	**15,201**	**9387**	**5107**

Supplementary Table 2

Term	Overlap	P-value	Adjusted P-value	Odds Ratio	Cluster
GOBP RESPONSE TO IONIZING RADIATION	28 / 144	1.79e-07	1.88e-04	3.55	Groups III/IV/V (Other tissues)
GOBP CELLULAR RESPONSE TO DNA DAMAGE STIMULUS	108 / 868	5.97e-11	3.13e-07	2.14	Groups III/IV/V (Other tissues)
GOBP DNA REPAIR	75 / 584	1.52e-08	3.30e-05	2.19	Groups III/IV/V (Other tissues)
GOBP MONOSACCHARIDE METABOLIC PROCESS	29 / 256	1.91e-06	2.11e-03	2.96	Groups I/II (Brain)
GOBP NCRNA METABOLIC PROCESS	55 / 613	1.95e-07	8.56e-04	2.31	Groups I/II (Brain)
GOBP NCRNA PROCESSING	42 / 433	7.49e-07	1.10e-03	2.50	Groups I/II (Brain)
GOBP DNA INTEGRITY CHECKPOINT SIGNALING	23 / 132	1.49e-05	4.27e-03	3.10	Groups III/IV/V (Other tissues)
GOBP CARBOHYDRATE METABOLIC PROCESS	53 / 594	3.89e-07	8.56e-04	2.29	Groups I/II (Brain)
GOBP DNA METABOLIC PROCESS	115 / 1043	1.89e-08	3.30e-05	1.86	Groups III/IV/V (Other tissues)
GOBP RNA SPLICING	59 / 463	6.93e-07	5.14e-04	2.15	Groups III/IV/V (Other tissues)
GOBP CELL CYCLE CHECKPOINT SIGNALING	29 / 188	1.44e-05	4.27e-03	2.68	Groups III/IV/V (Other tissues)
GOBP DOUBLE STRAND BREAK REPAIR	42 / 303	3.38e-06	1.61e-03	2.36	Groups III/IV/V (Other tissues)

bioRxiv preprint doi: https://doi.org/10.1101/2025.04.06.646893; this version posted April 8, 2025. The copyright holder for this preprint (which was not certified by peer review) is the author/funder, who has granted bioRxiv a license to display the preprint in perpetuity. It is made available under aCC-BY 4.0 International license.

GOBP HISTONE MODIFICATION	60 / 478	9.27e-07	5.40e-04	2.12	Groups III/IV/V (Other tissues)
GOBP ORGANELLE ASSEMBLY	108 / 993	1.07e-07	1.40e-04	1.82	Groups III/IV/V (Other tissues)
GOBP POSITIVE REGULATION OF RESPONSE TO DNA DAMAGE STIMULUS	26 / 167	3.33e-05	7.29e-03	2.71	Groups III/IV/V (Other tissues)
GOBP SIGNAL TRANSDUCTION IN RESPONSE TO DNA DAMAGE	28 / 184	2.59e-05	6.06e-03	2.63	Groups III/IV/V (Other tissues)
GOBP CYTOSKELETON ORGANIZATION	53 / 1509	2.90e-06	8.76e-03	2.16	Group VI (Testis)
GOBP MRNA METABOLIC PROCESS	86 / 767	6.40e-07	5.14e-04	1.87	Groups III/IV/V (Other tissues)
GOBP RNA LOCALIZATION	29 / 197	3.52e-05	7.40e-03	2.53	Groups III/IV/V (Other tissues)
GOBP REGULATION OF DNA METABOLIC PROCESS	64 / 539	2.75e-06	1.45e-03	1.99	Groups III/IV/V (Other tissues)
GOBP DNA RECOMBINATION	43 / 328	1.09e-05	3.90e-03	2.21	Groups III/IV/V (Other tissues)
GOBP NCRNA METABOLIC PROCESS	70 / 613	3.82e-06	1.67e-03	1.90	Groups III/IV/V (Other tissues)
GOBP CELLULAR RESPONSE TO DNA DAMAGE STIMULUS	66 / 868	4.46e-06	3.93e-03	1.93	Groups I/II (Brain)
GOBP CILIUM ORGANIZATION	51 / 421	1.63e-05	4.27e-03	2.02	Groups III/IV/V (Other tissues)
GOBP POSITIVE REGULATION OF DNA METABOLIC PROCESS	39 / 303	4.17e-05	8.37e-03	2.17	Groups III/IV/V (Other tissues)
GOBP CELLULAR RESPONSE TO STRESS	182 / 1973	7.83e-07	5.14e-04	1.53	Groups III/IV/V (Other tissues)
GOBP CELL PROJECTION ASSEMBLY	68 / 609	1.11e-05	3.90e-03	1.85	Groups III/IV/V (Other tissues)
GOBP MRNA PROCESSING	57 / 497	2.66e-05	6.06e-03	1.90	Groups III/IV/V (Other tissues)

bioRxiv preprint doi: https://doi.org/10.1101/2025.04.06.646893; this version posted April 8, 2025. The copyright holder for this preprint (which was not certified by peer review) is the author/funder, who has granted bioRxiv a license to display the preprint in perpetuity. It is made available under aCC-BY 4.0 International license.

Supplementary Table 3

GWAS trait	Loci	u-sQTL colocalize	Percentage colocalized
Age when finished full-time education	307	27	8.79%
Asthma childhood onset	109	9	8.26%
Atopic eczema	70	1	1.43%
Atrial fibrillation	111	4	3.60%
Basal cell carcinoma	94	7	7.45%
Bipolar disorder	65	11	16.92%
Breast cancer	168	12	7.14%
Chronic obstructive pulmonary disease	432	33	7.64%
Coronary artery disease	221	22	9.95%
Crohns disease	97	6	6.19%
Dupuytrens disease	64	4	6.25%
Heart failure	42	5	11.90%
Hypothyroidism	151	12	7.95%
Inflammatory bowel disease	141	12	8.51%
Multiple sclerosis	76	11	14.47%
Myocardial infarction	84	8	9.52%
Rheumatoid arthritis	76	0	0%
Schizophrenia	202	17	8.42%
Ulcerative colitis	62	5	8.06%
Visceral adipose tissue measurement	325	30	9.23%
**Total**	**2897**	**236**	**8.15%**

bioRxiv preprint doi: https://doi.org/10.1101/2025.04.06.646893; this version posted April 8, 2025. The copyright holder for this preprint (which was not certified by peer review) is the author/funder, who has granted bioRxiv a license to display the preprint in perpetuity. It is made available under aCC-BY 4.0 International license.

Supplementary Table 4

chr	Gene ID	Gene name
1	ENSG00000076356	*PLXNA2*
1	ENSG00000117322	*CR2*
1	ENSG00000203710	*CR1*
2	ENSG00000011523	*CEP68*
2	ENSG00000071051	*NCK2*
2	ENSG00000072135	*PTPN18*
2	ENSG00000072163	*LIMS2*
2	ENSG00000119147	*ECRG4*
2	ENSG00000123636	*BAZ2B*
2	ENSG00000136710	*CCDC115*
2	ENSG00000136717	*BIN1*
2	ENSG00000136731	*UGGT1*
2	ENSG00000138380	*CARF*
2	ENSG00000138442	*WDR12*
2	ENSG00000143951	*WDPCP*
2	ENSG00000143952	*VPS54*
2	ENSG00000143995	*MEIS1*
2	ENSG00000144426	*NBEAL1*
2	ENSG00000163161	*ERCC3*
2	ENSG00000163596	*ICA1L*
2	ENSG00000168918	*INPP5D*
3	ENSG00000163655	*GMPS*
3	ENSG00000169282	*KCNAB1*
3	ENSG00000174953	*DHX36*
4	ENSG00000087008	*ACOX3*
4	ENSG00000109452	*INPP4B*
4	ENSG00000109458	*GAB1*
4	ENSG00000127415	*IDUA*
4	ENSG00000145214	*DGKQ*

bioRxiv preprint doi: https://doi.org/10.1101/2025.04.06.646893; this version posted April 8, 2025. The copyright holder for this preprint (which was not certified by peer review) is the author/funder, who has granted bioRxiv a license to display the preprint in perpetuity. It is made available under aCC-BY 4.0 International license.

4	ENSG00000174227	*PIGG*
4	ENSG00000215375	*MYL5*
5	ENSG00000050748	*MAPK9*
5	ENSG00000051596	*THOC3*
5	ENSG00000113119	*TMCO6*
5	ENSG00000113194	*FAF2*
5	ENSG00000113249	*HAVCR1*
5	ENSG00000120725	*SIL1*
5	ENSG00000127022	*CANX*
5	ENSG00000135077	*HAVCR2*
5	ENSG00000145715	*RASA1*
5	ENSG00000145850	*TIMD4*
5	ENSG00000145901	*TNIP1*
5	ENSG00000146090	*RASGEF1C*
5	ENSG00000146094	*DOK3*
5	ENSG00000155508	*CNOT8*
5	ENSG00000183258	*DDX41*
5	ENSG00000204962	*PCDHA8*
5	ENSG00000285476	*AC139491.7*
6	ENSG00000146122	*DAAM2*
6	ENSG00000249853	*HS3ST5*
7	ENSG00000005020	*SKAP2*
7	ENSG00000006530	*AGK*
7	ENSG00000106261	*ZKSCAN1*
7	ENSG00000106327	*TFR2*
7	ENSG00000106351	*AGFG2*
7	ENSG00000106460	*TMEM106B*
7	ENSG00000146648	*EGFR*
7	ENSG00000153814	*JAZF1*
7	ENSG00000157800	*SLC37A3*
7	ENSG00000159784	*FAM131B*
7	ENSG00000159840	*ZYX*

bioRxiv preprint doi: https://doi.org/10.1101/2025.04.06.646893; this version posted April 8, 2025. The copyright holder for this preprint (which was not certified by peer review) is the author/funder, who has granted bioRxiv a license to display the preprint in perpetuity. It is made available under aCC-BY 4.0 International license.

7	ENSG00000185899	*TAS2R60*
7	ENSG00000197037	*ZSCAN25*
7	ENSG00000257923	*CUX1*
8	ENSG00000012232	*EXTL3*
8	ENSG00000070756	*PABPC1*
8	ENSG00000104517	*UBR5*
8	ENSG00000120885	*CLU*
8	ENSG00000120899	*PTK2B*
8	ENSG00000168077	*SCARA3*
8	ENSG00000174226	*SNX31*
8	ENSG00000186106	*ANKRD46*
8	ENSG00000186918	*ZNF395*
10	ENSG00000048740	*CELF2*
10	ENSG00000059573	*ALDH18A1*
10	ENSG00000095585	*BLNK*
10	ENSG00000095637	*SORBS1*
10	ENSG00000107679	*PLEKHA1*
10	ENSG00000108219	*TSPAN14*
10	ENSG00000122870	*BICC1*
10	ENSG00000122873	*CISD1*
10	ENSG00000148429	*USP6NL*
10	ENSG00000151150	*ANK3*
10	ENSG00000151151	*IPMK*
10	ENSG00000155229	*MMS19*
10	ENSG00000166033	*HTRA1*
10	ENSG00000185737	*NRG3*
11	ENSG00000030066	*NUP160*
11	ENSG00000073921	*PICALM*
11	ENSG00000134569	*LRP4*
11	ENSG00000137642	*SORL1*
11	ENSG00000149196	*HIKESHI*
11	ENSG00000149201	*CCDC81*

bioRxiv preprint doi: https://doi.org/10.1101/2025.04.06.646893; this version posted April 8, 2025. The copyright holder for this preprint (which was not certified by peer review) is the author/funder, who has granted bioRxiv a license to display the preprint in perpetuity. It is made available under aCC-BY 4.0 International license.

11	ENSG00000150672	*DLG2*
11	ENSG00000150687	*PRSS23*
11	ENSG00000165915	*SLC39A13*
11	ENSG00000166801	*FAM111A*
11	ENSG00000172409	*CLP1*
12	ENSG00000089060	*SLC8B1*
12	ENSG00000089127	*OAS1*
12	ENSG00000089169	*RPH3A*
12	ENSG00000123066	*MED13L*
12	ENSG00000135094	*SDS*
12	ENSG00000139405	*RITA1*
12	ENSG00000173064	*HECTD4*
12	ENSG00000186815	*TPCN1*
14	ENSG00000012963	*UBR7*
14	ENSG00000066427	*ATXN3*
14	ENSG00000100478	*AP4S1*
14	ENSG00000100599	*RIN3*
14	ENSG00000100605	*ITPK1*
14	ENSG00000100883	*SRP54*
14	ENSG00000100890	*PRORP*
14	ENSG00000133958	*UNC79*
14	ENSG00000140090	*SLC24A4*
14	ENSG00000165943	*MOAP1*
15	ENSG00000081014	*AP4E1*
15	ENSG00000138613	*APH1B*
15	ENSG00000140455	*USP3*
15	ENSG00000157470	*FAM81A*
15	ENSG00000180304	*OAZ2*
16	ENSG00000064270	*ATP2C2*
16	ENSG00000103175	*WFDC1*
16	ENSG00000135686	*KLHL36*
16	ENSG00000140943	*MBTPS1*

bioRxiv preprint doi: https://doi.org/10.1101/2025.04.06.646893; this version posted April 8, 2025. The copyright holder for this preprint (which was not certified by peer review) is the author/funder, who has granted bioRxiv a license to display the preprint in perpetuity. It is made available under aCC-BY 4.0 International license.

16	ENSG00000197943	*PLCG2*
17	ENSG00000011143	*MKS1*
17	ENSG00000030582	*GRN*
17	ENSG00000067596	*DHX8*
17	ENSG00000073969	*NSF*
17	ENSG00000178852	*EFCAB13*
18	ENSG00000101695	*RNF125*
18	ENSG00000118276	*B4GALT6*
18	ENSG00000134758	*RNF138*
18	ENSG00000141441	*GAREM1*
19	ENSG00000007047	*MARK4*
19	ENSG00000010310	*GIPR*
19	ENSG00000011304	*PTBP1*
19	ENSG00000011422	*PLAUR*
19	ENSG00000011478	*QPCTL*
19	ENSG00000011485	*PPP5C*
19	ENSG00000012061	*ERCC1*
19	ENSG00000042753	*AP2S1*
19	ENSG00000064687	*ABCA7*
19	ENSG00000065000	*AP3D1*
19	ENSG00000065268	*WDR18*
19	ENSG00000073050	*XRCC1*
19	ENSG00000076928	*ARHGEF1*
19	ENSG00000079313	*REXO1*
19	ENSG00000079385	*CEACAM1*
19	ENSG00000079432	*CIC*
19	ENSG00000089847	*ANKRD24*
19	ENSG00000090372	*STRN4*
19	ENSG00000090554	*FLT3LG*
19	ENSG00000099804	*CDC34*
19	ENSG00000099821	*POLRMT*
19	ENSG00000099864	*PALM*

bioRxiv preprint doi: https://doi.org/10.1101/2025.04.06.646893; this version posted April 8, 2025. The copyright holder for this preprint (which was not certified by peer review) is the author/funder, who has granted bioRxiv a license to display the preprint in perpetuity. It is made available under aCC-BY 4.0 International license.

19	ENSG00000104783	*KCNN4*
19	ENSG00000104852	*SNRNP70*
19	ENSG00000104853	*CLPTM1*
19	ENSG00000104859	*CLASRP*
19	ENSG00000104866	*PPP1R37*
19	ENSG00000104884	*ERCC2*
19	ENSG00000104885	*DOT1L*
19	ENSG00000104936	*DMPK*
19	ENSG00000104967	*NOVA2*
19	ENSG00000104973	*MED25*
19	ENSG00000105255	*FSD1*
19	ENSG00000105281	*SLC1A5*
19	ENSG00000105287	*PRKD2*
19	ENSG00000105321	*CCDC9*
19	ENSG00000105372	*RPS19*
19	ENSG00000105383	*CD33*
19	ENSG00000105402	*NAPA*
19	ENSG00000105426	*PTPRS*
19	ENSG00000105429	*MEGF8*
19	ENSG00000105486	*LIG1*
19	ENSG00000105497	*ZNF175*
19	ENSG00000105501	*SIGLEC5*
19	ENSG00000105732	*ZNF574*
19	ENSG00000105737	*GRIK5*
19	ENSG00000115266	*APC2*
19	ENSG00000118162	*KPTN*
19	ENSG00000121289	*CEP89*
19	ENSG00000124440	*HIF3A*
19	ENSG00000124449	*IRGC*
19	ENSG00000124459	*ZNF45*
19	ENSG00000125743	*SNRPD2*
19	ENSG00000125744	*RTN2*

bioRxiv preprint doi: https://doi.org/10.1101/2025.04.06.646893; this version posted April 8, 2025. The copyright holder for this preprint (which was not certified by peer review) is the author/funder, who has granted bioRxiv a license to display the preprint in perpetuity. It is made available under aCC-BY 4.0 International license.

19	ENSG00000125746	*EML2*
19	ENSG00000125755	*SYMPK*
19	ENSG00000129932	*DOHH*
19	ENSG00000130005	*GAMT*
19	ENSG00000130202	*NECTIN2*
19	ENSG00000130203	*APOE*
19	ENSG00000130208	*APOC1*
19	ENSG00000130751	*NPAS1*
19	ENSG00000131115	*ZNF227*
19	ENSG00000131116	*ZNF428*
19	ENSG00000141873	*SLC39A3*
19	ENSG00000141905	*NFIC*
19	ENSG00000142002	*DPP9*
19	ENSG00000142530	*FAM71E1*
19	ENSG00000159917	*ZNF235*
19	ENSG00000160014	*CALM3*
19	ENSG00000160570	*DEDD2*
19	ENSG00000161091	*MFSD12*
19	ENSG00000161249	*DMKN*
19	ENSG00000167470	*MIDN*
19	ENSG00000167637	*ZNF283*
19	ENSG00000167671	*UBXN6*
19	ENSG00000167680	*SEMA6B*
19	ENSG00000172006	*ZNF554*
19	ENSG00000174917	*MICOS13*
19	ENSG00000176490	*DIRAS1*
19	ENSG00000177051	*FBXO46*
19	ENSG00000178150	*ZNF114*
19	ENSG00000178386	*ZNF223*
19	ENSG00000179954	*SSC5D*
19	ENSG00000181027	*FKRP*
19	ENSG00000182087	*TMEM259*

bioRxiv preprint doi: https://doi.org/10.1101/2025.04.06.646893; this version posted April 8, 2025. The copyright holder for this preprint (which was not certified by peer review) is the author/funder, who has granted bioRxiv a license to display the preprint in perpetuity. It is made available under aCC-BY 4.0 International license.

19	ENSG00000185800	*DMWD*
19	ENSG00000186026	*ZNF284*
19	ENSG00000187116	*LILRA5*
19	ENSG00000187244	*BCAM*
19	ENSG00000188624	*IGFL3*
19	ENSG00000197380	*DACT3*
19	ENSG00000197405	*C5AR1*
19	ENSG00000214456	*PLIN5*
19	ENSG00000224916	*APOC4-APOC2*
19	ENSG00000234465	*PINLYP*
19	ENSG00000234906	*APOC2*
19	ENSG00000256294	*ZNF225*
19	ENSG00000267001	*AC006538.2*
19	ENSG00000267022	*AC067968.1*
19	ENSG00000267385	*AC011498.4*
19	ENSG00000267467	*APOC4*
19	ENSG00000267508	*ZNF285*
19	ENSG00000267680	*ZNF224*
19	ENSG00000268361	*L34079.1*
19	ENSG00000269469	*AC010619.1*
19	ENSG00000278318	*ZNF229*
20	ENSG00000087510	*TFAP2C*
20	ENSG00000087589	*CASS4*

bioRxiv preprint doi: https://doi.org/10.1101/2025.04.06.646893; this version posted April 8, 2025. The copyright holder for this preprint (which was not certified by peer review) is the author/funder, who has granted bioRxiv a license to display the preprint in perpetuity. It is made available under aCC-BY 4.0 International license.

Supplementary Table 5

**Gene ID**	**Gene name**	**Brain regions**	**PTWAS_PVAL**	**Colocalization**
ENSG00000073921	*PICALM*	AC,DLPFC,PCC	3.75E-30	eQTL & sQTL & GWAS
ENSG00000130208	*APOC1*	AC,DLPFC,PCC	1.02E-24	eQTL & GWAS
ENSG00000127415	*IDUA*	AC,DLPFC,PCC	4.41E-19	No Colocalization
ENSG00000108219	*TSPAN14*	AC,DLPFC,PCC	1.77E-14	eQTL & sQTL & GWAS
ENSG00000087589	*CASS4*	AC	1.39E-13	eQTL & sQTL & GWAS
ENSG00000072135	*PTPN18*	AC,DLPFC,PCC	4.70E-13	No Colocalization
ENSG00000165915	*SLC39A13*	DLPFC	1.11E-08	eQTL & GWAS
ENSG00000087008	*ACOX3*	AC,DLPFC	3.45E-08	No Colocalization
ENSG00000163596	*ICA1L*	AC,DLPFC,PCC	3.23E-07	No Colocalization
ENSG00000104885	*DOT1L*	AC,DLPFC,PCC	6.28E-07	No Colocalization
ENSG00000138442	*WDR12*	AC,PCC	8.83E-07	No Colocalization
ENSG00000136717	*BIN1*	AC,DLPFC,PCC	1.09E-06	eQTL & GWAS
ENSG00000167671	*UBXN6*	AC,DLPFC	1.87E-06	No Colocalization
ENSG00000073969	*NSF*	AC	5.94E-06	No Colocalization
ENSG00000064687	*ABCA7*	AC,DLPFC	1.50E-05	No Colocalization
ENSG00000104884	*ERCC2*	AC,DLPFC,PCC	0.00014341	eQTL & GWAS
ENSG00000141441	*GAREM1*	AC,DLPFC	0.00038832	No Colocalization
